# Ten seconds of my nights: Exploring methods to measure brightness, loudness and attendance and their associations with alcohol use from video clips

**DOI:** 10.1371/journal.pone.0250443

**Published:** 2021-04-28

**Authors:** Florian Labhart, Skanda Muralidhar, Benoit Massé, Lakmal Meegahapola, Emmanuel Kuntsche, Daniel Gatica-Perez

**Affiliations:** 1 Centre for Alcohol Policy Research, La Trobe University, Melbourne, Australia; 2 Idiap Research Institute, Martigny, Switzerland; 3 Addiction Switzerland, Research Institute, Lausanne, Switzerland; 4 Ecole Polytechnique Fédérale de Lausanne (EPFL), Lausanne, Switzerland; University of Queensland, AUSTRALIA

## Abstract

**Introduction:**

Most evidence on associations between alcohol use behaviors and the characteristics of its social and physical context is based on self-reports from study participants and, thus, only account for their subjective impressions of the situation. This study explores the feasibility of obtaining alternative measures of loudness, brightness, and attendance (number of people) using 10-second video clips of real-life drinking occasions rated by human annotators and computer algorithms, and explores the associations of these measures with participants’ choice to drink alcohol or not.

**Methods:**

Using a custom-built smartphone application, 215 16-25-year-olds documented characteristics of 2,380 weekend night drinking events using questionnaires and videos. Ratings of loudness, brightness, and attendance were obtained from three sources, namely in-situ participants’ ratings, video-based annotator ratings, and video-based computer algorithm ratings. Bivariate statistics explored differences in ratings across sources. Multilevel logistic regressions assessed the associations of contextual characteristics with alcohol use. Finally, model fit indices and cross-validation were used to assess the ability of each set of contextual measures to predict participants’ alcohol use.

**Results:**

Raw ratings of brightness, loudness and attendance differed slightly across sources, but were all correlated (*r* = .21 to .82, all *p* < .001). Participants rated bars/pubs as being louder (Cohen’s *d* = 0.50 [95%-CI: 0.07–0.92]), and annotators rated private places as darker (*d* = 1.21 [95%-CI: 0.99–1.43]) when alcohol was consumed than when alcohol was not consumed. Multilevel logistic regressions showed that drinking in private places was more likely in louder (OR_participants_ = 1.74 [CI: 1.31–2.32]; OR_annotators_ = 3.22 [CI: 2.06–5.03]; OR_algorithm_ = 2.62 [CI: 1.83–3.76]), more attended (OR_participants_ = 1.10 [CI: 1.03–1.18]; OR_algorithm_ = 1.19 [CI: 1.07–1.32]) and darker (OR = 0.64 [CI: 0.44–0.94]) situations. In commercial venues, drinking was more likely in darker (OR_participants_ = 0.67 [CI: 0.47–0.94]; OR_annotators_ = 0.53 [CI: 0.33–0.85]; OR_algorithm_ = 0.58 [CI: 0.37–0.88]) and louder (OR_participants_ = 1.40 [CI: 1.02–1.92]; OR_algorithm_ = 2.45 [CI: 1.25–4.80]) places. Higher inference accuracies were found for the models based on the annotators’ ratings (80% to 84%) and the algorithms’ ratings (76% to 86%) than on the participants’ ratings (69% to 71%).

**Conclusions:**

Several contextual characteristics are associated with increased odds of drinking in private and commercial settings, and might serve as a basis for the development of prevention measures. Regarding assessment of contextual characteristics, annotators and algorithms might serve as appropriate substitutes of participants’ in-situ impressions for correlational and regression analyses despite differences in raw ratings. Collecting contextual data by means of sensors or media files is recommended for future research.

## Introduction

Every drinking occasion takes place in a given physical and social context [1–3]. To date, most evidence on the associations between contextual characteristics and alcohol use are based on the participants’ subjective impressions of the drinking occasion. Yet, actors in a given situation may have problems recalling or even noticing relevant contextual characteristics. Capturing contextual characteristics from an external observer-like viewpoint is therefore important to obtain more objective and ecologically valid data on contextual correlates of alcohol use. However, collecting such information is methodologically challenging. Using a dataset of 10-second video clips recoded in real-life situations, the purposes of this paper are to explore differences in the assessment of brightness, loudness, and attendance by human participants (in-situ reports), human annotators (video coding), and computer algorithms (video analysis) in various contexts, and to examine how these contextual characteristics are associated with the consumption of alcohol. Alongside the development of wearable technologies and sensors to unobtrusively collect data on people’s real-life behaviors [4, 5], this study stands as a first exploration of whether and how participants’ reports of the context could be replaced by similar measures from other sources.

### Contextual correlates of alcohol use: Evidence from scattered perspectives

Our understanding of peoples’ behaviors in their immediate environment largely depends on the source of the collected data. In sociology, a distinction is traditionally made between the people involved in a situation (the insiders) and those who observe the former (the outsiders). The insiders are the actors–or ‘subjects’–in the situation and can provide knowledge permeated by the history and symbolic meaning of their current situation [6]. In contrast, the outsiders–or observers–are blinded to such specificities and rather consider the situation as an ‘object’ of observation [6]. Collecting information from both perspectives–subjective experience of the participants and objective observation–are therefore ideal to gain a comprehensive picture of a given drinking occasion [7, 8]. However, this has not been implemented in alcohol research and most studies only reflect one perspective at a time.

Self-reports are typically used to identify which characteristics of the context, perceived subjectively by the participants, are associated with alcohol use. Cross-sectional retrospective surveys [e.g., 9–11] and diary-based methods, such as ecological momentary assessment (EMA) [e.g., 12–15], concur with the observation that people are more likely to drink alcohol in specific locations and in the presence of large groups of friends. For example, Canadian students reported consuming more alcohol per occasion when they were at a party (as opposed to any other drinking occasions), in a bar or at home than in a restaurant, as well as with large groups of friends [10]. Similarly, young Swiss adults were found to drink higher amounts of alcohol when drinking occasions started in private places before going out [14], and in the presence of a higher number of friends [12, 13]. Additionally, evidence from qualitative interviews revealed that young people take control over their experience of drunkenness in private settings by staging the atmosphere, notably with selected music styles and lighting [16, 17]. However, a major drawback of self-reports is the risk of omissions and recall errors, as respondents are known to forget details of their experiences within a couple of days [18, 19].

In-situ observations and experiments are generally used to document characteristics of drinking settings and their association with alcohol use from an external observer viewpoint [20–22]. For instance, in-bar experiments manipulating levels of music loudness revealed that patrons’ drinking pace and amounts increased in louder environments [23, 24]. To explain this phenomenon, the authors argued that high sound levels create a high level of arousal among patrons, who enhance their behavioral response toward the stimulus [24, 25]. While music at moderate sound levels eases the socialization process in pubs and nightclubs [26], loud music likely impedes conversation and likely increases patrons’ drinking pace. Besides this, observational studies have shown that bar patrons’ intoxication levels are also associated with promotion of soft or energy drinks, poor washroom facilities or the presence of a dance floor, but not with environmental brightness or perceived music sound level [27]. A major advantage of in-situ sensing (e.g. with sound level meters) and observation is that these methods provide standardized measures that can be compared across locations and times. However, such methods cannot easily be used outside of publicly accessible locations and cannot follow individuals when they change locations.

Overall, this body of findings provides concurring evidence that particular characteristics of the social (e.g., the size of the drinking group) and physical context (e.g., the type of location and loudness level) can be associated with peoples’ drinking behaviors, both when documented by participants and observers. However, an important limitation of the existing literature, besides the fact that each study only accounted for the perception of either the participants or the observers, is the limited types of locations investigated, especially with in-situ observations. While most evidence has been collected in commercial venues (bars and nightclubs), little is known about the drinking context characteristics of other public spaces, such as parks, streets, and means of transportation, as well as in private settings.

### Capturing contextual characteristics with 10-second video clips

The present study elaborates on the technological developments of smartphones to explore the feasibility of assessing the physical and social characteristics of various drinking settings from both actors’ and observers’ perspectives. As part of a larger study on young adults’ nightlife and drinking behaviors, we developed a smartphone application that is able to collect various behavioral and contextual data in real time by means of questionnaires, built-in sensors, pictures, and short video clips [28]. Among other things, participants described the perceived levels of brightness, loudness, and the number of people present every time they had an alcoholic or a non-alcoholic drink. Additionally, for every drink in a new location, they were requested to record a 10-second panoramic video clip of the surrounding context, to be later annotated by research assistants and analyzed via computer algorithms.

Video clips were requested to capture data that could not be obtained by the smartphones built-in sensors and that represented an additional perspective other than the participants’ subjective perception of the situation. Our intention in requesting a 360-degree panorama of the situation was to obtain a comprehensive audio-visual representation, similar to the one that could be obtained from an external observer’s perspective [7, 8]. Additionally, given the videos were recorded at the same time and place as the consumed beverage, they provided data with a very high degree of ecological validity. Lastly, an important asset of the video clips is the ability to document contextual elements that participants may be unaware of, or do not pay attention to, and would therefore not report accurately a posteriori.

Exploratory analyses of the videos content revealed that typical levels of brightness varied across location, with bars and nightclubs being darker and louder than other public spaces and private places [5]. Participants’ ratings of brightness and loudness correlated poorly to moderately with those of annotators based on the videos. In contrast, annotators’ ratings were globally consistent with those measured by computer algorithms [5]. These findings suggest that the video content can effectively be used to assess contextual characteristics and that annotators and computer algorithms rated some ambiance attributes differently compared to the participants. However, limitations in analytic approach, such as the absence of a systematic annotation procedure (i.e., several annotators worked on distinct subsets of videos) and the absence of conversion of loudness and brightness levels to the human perception specificities (i.e., human perception of loudness and brightness is not linear; see Measures section below) called for an improved approach.

For the present study, five independent annotators annotated the entire collection of 843 video clips, and the brightness and loudness extraction algorithms were redeveloped to match human perception specificities. Additionally, we extended the analytic frame to the social context, namely the number of people appearing in the videos, as the presence and number of people was frequently highlighted as a core component of the context by previous research [10, 12, 13]. Although other momentary circumstances were shown to be associated with participants’ choice to drink alcohol or not (e.g., event-specific drinking motives and expectancies [29, 30], parental supervision [31, 32]), we selected characteristics that could be measured in a comparable way by participants, annotators, and computer algorithms. Given that algorithms are not as capable as humans to identify social bounds or cognitions, we restricted the focus on factual characteristics of the physical and social context.

### Study aims

The Youth@Night study was conceived as a natural experiment in which participants documented drinking situations, in various settings, where they could freely choose to drink alcohol or not. Given the public health burden of heavy alcohol use among young people on weekend nights [33, 34], the main aim of this study was to identify which contextual characteristics were associated with participants’ choice to drink alcohol or not. Additionally, given the unique opportunity to compare three measures of the same contextual characteristics from different data sources, the study also aimed to explore the specificities of the data from each source and their ability to predict participants’ choice to drink alcohol or not in various contexts.

The first part of the analysis explored the distributions and bivariate associations of the raw ratings of brightness, loudness, and attendance provided by participants, annotators, and computer algorithms.

The second part investigated how levels of brightness, loudness, and attendance measured by each source vary depending on whether the participants were drinking an alcoholic or a non-alcoholic drink in seven different types of locations including bars, nightclubs, restaurants, public spaces, and homes.

The third part aimed to identify which of the three contextual characteristics were associated with participants’ choice to drink alcohol or not, above and beyond each other, using series of logistic regression models.

The last part of the analysis evaluated the ability of each set of contextual measures from each of the sources to predict participants’ choice to drink alcohol or not, by comparing the percentage of variance explained, the goodness of fit, and the inference accuracy of each model.

## Materials and methods

### Study design

Participants were recruited in the streets of the two major nightlife hubs in Switzerland (Lausanne and Zurich) between 9pm and midnight on Friday and Saturday nights in the first three weekends of September 2014. In defined nightlife areas, research assistants approached every nth person crossing a ‘virtual line’ on the street [35]. Eligibility criteria were being aged between 16 and 25, owning an Android smartphone on which the Youth@Night app could be installed, having consumed alcohol at least once in the past month (legal drinking age is 16 for beer and wine in Switzerland), and having been out in the city at least twice in the past month. After explaining the aim and procedure of the study, recruiters recorded volunteers’ email address, and volunteers then automatically received an email containing a link to the study website and the online consent form. After signing the consent form and completing the baseline questionnaire, participants installed the Youth@Night application on their smartphone. This app was specifically developed to record various aspects of the participants’ Friday and Saturday nights, including the types of drinks consumed and the social and physical characteristics of locations attended over seven consecutive weekends, using questionnaires, pictures, video clips, and sensors [28]. The Lausanne and Zurich Cantonal Ethics Committees for Research on Human Beings (protocol 145/14) approved the study and did not request consent from parents of participants under the age of 18.

### Extraction of audio and visual cues from the videos

After the fieldwork, we developed an online annotation task to extract visual and audio cues from the videos recorded by the participants [36]. Five independent annotators watched, in a random order, the entire set of videos and annotated the type of location, the loudness and brightness levels, the number of people visible, and other situational cues (e.g., ongoing activities, people’s reaction to being filmed). After a training session, annotators completed the annotation task at their own pace over two months using their computer.

### Samples of participants and situations

In total, 3,092 people were approached in the two cities. Of those, 1,119 (36.2%) did not have an Android smartphone, 859 (27.8%) were not interested in participating in the study and 233 (7.5%) were outside the required age range of 16 to 25. Of the 881 who agreed to participate, 629 (71.4%) signed the online consent form, 367 completed the baseline questionnaire (41.7%) and 241 documented their nights using the smartphone app (27.4%) [35]. The sample of 241 participants (mean age = 19.0 [SD = 2.4]; 46.5% women) was slightly younger than the rest of the eligible pool of passers-by approached on the streets (mean age = 19.6 [SD = 2.8]; *t* = 2.76, *p* = .006) but similar in terms of gender ratio (41.2% women; χ^2^ = 2.24, *p* = .134).

Participants documented 2,420 drinking situations, each with a picture of the drink and a description of the context, 1,394 with labels of the locations, and 843 with video clips of the context (see Measures section below for the full sequence of questionnaires). While preparing the data for the analysis, we assigned the type of location to 987 additional drinking situations based on the sequence of events during the night, the GPS coordinates of the location and contextual cues visible in the background of drink pictures, and excluded 18 situations because the related videos were entirely black and silent. The analyses were conducted on a total sample of 2,358 drinking situations documented with data on the drink and the location, and 825 with a video. These situations were reported by 210 participants who were slightly older than the rest of the participants who installed the app (mean age = 19.2, SD = 2.4, *t* = -2.1; *p* = .037) but similar in terms of gender ratio (47.1% women; χ^2^ = 0.07, *p* = .791). Half of the 2,358 situations were reported by 42 participants (20%).

### Measures

#### Participants

*Alcoholic versus non-alcoholic drinks*. From 8 p.m. until the end of the night, participants were requested to take a picture of their drink every time they had a new drink, and to label it as one of six types of alcoholic drinks (e.g., beer, wine, spirits, cocktails; coded as 1) or six types of non-alcoholic drinks (e.g., water, soda, energy drink, tea/coffee; coded as 0).

*Brightness and loudness*. After labeling the drink type, participants described the level of contextual brightness and loudness on a five-point Likert scale ranging from 0 (very low) to 4 (very high).

*Attendance*. In the same questionnaire, participants reported the type and number of people present around them by indicating how many of the following people were present: ‘partner or spouse’ (0 or 1), ‘family or relatives’ (answer options: increasing integers from 0 to 10, plus ‘more than 10’ [coded as 15]), ‘male friends or colleagues’ (same options), ‘female friends or colleagues’ (same options), and ‘other people’ (same options). All categories were summed up to represent the total number of people present.

*Locations type*. When having their first drink of the night, and each time they changed location later in the night, participants were asked to report the type of location they were at. Responses were recoded into the following categories: ‘bars/pubs,’ ‘nightclubs,’ ‘restaurants,’ ‘events and leisure’ (e.g. sport arenas, concerts, bowling), ‘public parks and streets’, ‘travelling’ (e.g. on trains, cars) and ‘private places’. Locations indicated by the participants were compared with those identified by the annotators from the videos (see below) and, in case of disagreement, latitudinal and longitudinal coordinates were checked to ensure the correct categorization of the location. For the last parts of the analysis, locations were categorized at the coarser level into commercial venues (bars/pubs, nightclubs, restaurants, and events/leisure), non-commercial public spaces (parks, streets, and travelling), and private places.

*Video clip*. After reporting the location type, participants were asked to take a 10-second video clip of the location. The following instructions were shown in the app before each video recording in order to accurately record contextual loudness, brightness, and ongoing activities and to collect standardized content for later processing: use landscape format (horizontal), generate a full view (360°) of the venue by slowly turning from left to right, take a video even if the scene is dark, and do not cover the microphone with the hand. Participants could skip the video if they did not feel comfortable or ready (i.e., not an appropriate moment, not feeling safe, forbidden in the location, someone objected to it). The rates of recording a video after having taken a picture were 30.6% of the cases in private places, 39.4% in commercial venues (32.3% in restaurants, 35.9% in bars, 50.0% in nightclubs) and 44.0% in public spaces (39.6% on the street and in parks, 56.5% while traveling).

*Drinks consumed*. The number of drinks consumed earlier in the night was obtained by summing up the total number of alcoholic drinks already reported by the participants [28]. The amount was converted into standard drinks of 10 grams of pure alcohol [37].

#### Annotators

*Brightness* and *loudness*. External annotators rated physical characteristics of the context along three dimensions, namely brightness, music loudness, and chatter loudness, using the same five-point Likert scale used by participants (0 = ‘very low’, 4 = ‘very high’). The maximum score of music loudness and chatter loudness was selected to represent the overall loudness. Intraclass correlation coefficients (ICC) showed an excellent level of agreement between the five annotators for both dimensions (ICC(2,k)_brightness_ = 0.948, and ICC(2,k)_loudness_ = 0.955 [38, 39]). To obtain a 5-point scale, as for participants, annotators’ ratings were aggregated as follows: if the majority of annotators (3 or more) agreed on one value, this value was selected, otherwise the mean of the 5 ratings was rounded to the closest integer. Compared to systematically selecting the mean of the 5 annotations, this method has the advantage of giving more importance to concordant answers and being less sensitive to outliers.

*Attendance*. Annotators were asked to indicate how many people appeared in the video (in addition to the phone holder) using the following answer options: ‘0’, ‘1’, ‘2–4’ (coded as 3), ‘5–10’ (7.5) and ‘more than 10’ (15). An excellent level of agreement was found between the five annotators (ICC(2,k)_attendance_ = 0.915). Given the linear nature of the measure, recoded scores were averaged across annotators.

#### Computer algorithms

*Brightness*. Total average brightness was obtained by averaging the intensity of each pixel in each frame of the video across all frames [40]. The obtained measure was then transformed into human perceived brightness, also called relative luminance [41], on a 0 to 100 scale using Glasser’s formula [42]. Finally, to allow comparison with the participants’ and annotators’ ratings, perceived brightness was rescaled to a 0 to 4 scale using 20-point increment cut-offs (0 to 19.9 = 0; 20 to 39.9 = 1, etc.).

*Loudness*. The total average loudness was obtained by averaging the temporally-smoothed instantaneous audio power across all frames in the video [5, 43, 44]. The obtained measure was then log-transformed to account for the exponential nature of sound measurements converted into decibels (dB) and adjusted to the standard human hearing ability [45, 46]. To allow comparisons with the participants’ and annotators’ ratings, loudness was rescaled to a 0 to 4 scale using the following ranges: less than 40 dB, 40–49 dB, 50–69 dB, 70–84 dB, and 85 dB or more.

*Attendance*. On each video frame, we used the YOLOv3 object detector [47]. YOLOv3 uses a 53-layer fully convolutional neural network trained on the 80 categories from the MS-COCO dataset [48] to find bounding boxes containing the category "person". Counting boxes allows the algorithm to count the number of people in each video frame. To avoid counting each person multiple times and to identify which boxes correspond to the same person in successive frames, we used the Deep-SORT tracker [49] that combines a geometric approach (position, size, and speed of a bounding box sequence) [50] with an appearance model (whether the content of bounding boxes look similar or not) [51]. Finally, we report the number of identity clusters according to the tracker as the number of people shown in the video.

### Analytic strategy

Descriptive statistics were first used to report the average levels of brightness, loudness and attendance from the participants’, annotators’ and algorithms’ perspective. Level of agreement between participants’, annotators’ and algorithms’ ratings of brightness, loudness, and attendance were illustrated using correspondence matrices and measured with Pearson’s correlations and paired-sample *t*-tests. Effect sizes of correlations and *t*-tests were adjusted for the effect of the drinking situations being nested within participants using Stata 14 [35]. Correlation coefficients under .40 were considered as ‘poor’, between .40 and .59 as ‘fair’, between .60 and .74 as ‘good’ and above .75 as ‘excellent’, following the guidelines by Cicchetti [39].

Secondly, descriptive statistics were used to calculate the average levels of brightness, loudness and attendance from participants’, annotators’ and algorithms’ perspectives for each type of location. The magnitude of the difference in levels of brightness, loudness, and attendance between situations with and without alcohol use were assessed using Cohen’s *d* estimates and confidence intervals. Effect sizes of 0.2 were considered as small, 0.5 as medium and 0.8 or more as large [52] and the effects were considered significant if the confidence interval did not contain ‘0’ [53].

Thirdly, a series of multilevel logistic regression models were estimated to investigate the mutually adjusted associations of contextual variables with the likelihood of drinking an alcoholic versus a non-alcoholic drink. Nine models were estimated to explore the location-specific associations of the context with alcohol, separately for each data source and the three main location types (i.e., commercial venues, public spaces, and private places; due to the small number of observations in some locations types, the models were estimated only at the coarser level) using the following formula: $y_{ij}=\beta_{0}{brightness}_{ij}+\beta_{1}{loudness}_{ij}+\beta_{2}{attendance}_{ij}+\beta_{3}{prior\_drinks}_{ij}+z_{ij}u_{j}+\epsilon_{ij}$; for *j* = 1,…, *n* participants, with participant *j* consisting of *i* = 1,…,*n*_*j*_ observations. Situation-level predictors also included the number of alcoholic drinks consumed earlier in the night because participants’ perception of their context might be altered by their level of inebriation. All models were estimated using the maximum likelihood robust (MLR) estimator to account for deviation from normal distribution using Stata 14 [35]. Reported effect sizes were odds ratios (OR) and 95%-confidence intervals.

Finally, the ability of each set of contextual measures to predict participants’ choice to drink alcohol or not were compared based on the percentage of variance explained, goodness of fit, and inference accuracy of the nine logistic regression models. These indices were computed using the following approaches:

- The percentage of variance explained was estimated using the McKelvey & Zavoina’s pseudo R-squared using the “fit_meologit_2lev” package for Stata [54]. In Table 3, we report the estimate based on the fixed effects only, which is a good estimate of how the model might fare in a different sample (in opposition to the estimate based on the fixed and random effects, which is a good estimate of how well the current model is calibrated to the current data).
- The goodness of fit was estimated using a variation of the Hosmer-Lemeshow test for multilevel logistic models in Stata [55]. We report the Hosmer-Lemeshow Chi-squared and *p*-value based on the fixed effects only (see rationale above), and a *p*-value below .05 indicates that the model is not a good fit.
- Inference accuracy was estimated using a two-class inference task, with "drinking alcohol" versus "drinking no alcohol" as a target classes, using python with scikitlearn and keras [56]. We trained and tested six models types: random forest classifier, naive Bayes, gradient boosting, XGboost, AdaBoost, and support vector machines (in this decreasing order of overall inference accuracies), and repeated each inference task for 10 iterations. Because of the data imbalance between the number of points for each class (i.e., situations with or without alcohol), we oversampled the minority class (i.e., the class with fewer data points) [57] in order to obtain balanced datasets in each iteration, and then, evaluated the models using 10-fold cross-validation [58]. In this paper, we report the results for random forest classifiers (RFs) which provided the highest accuracy values using n-tree values between 200 and 500 (results of the other model types to be obtained from authors upon request).

## Results

### Levels of brightness, loudness, and attendance per data source

**Fig 1:**
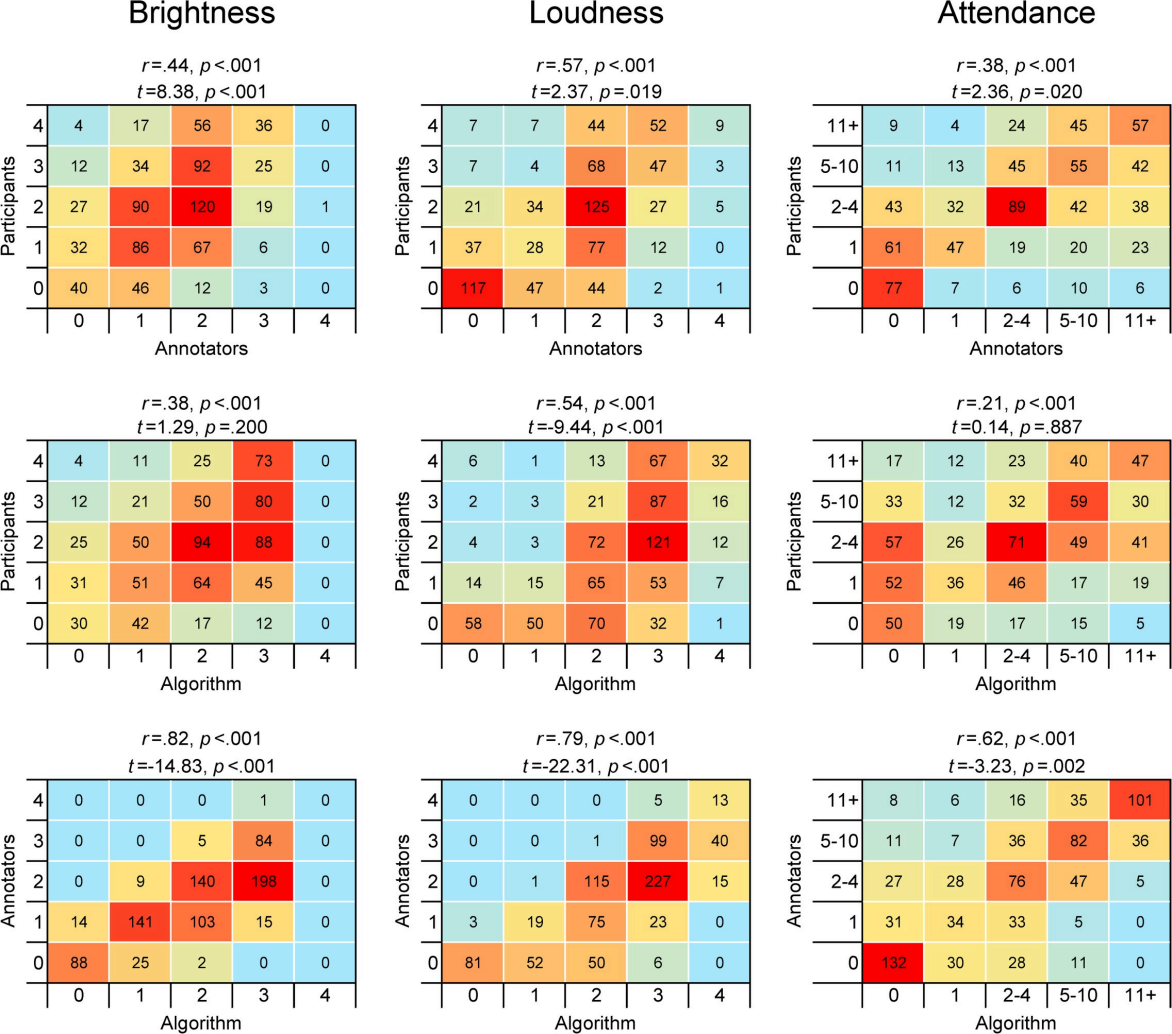
Correspondence matrix, Pearson’s correlations and mean difference tests of participants’, annotators’ and computer algorithms’ ratings of brightness, loudness and attendance in the 825 drinking situations.

As seen in Table 1 and Fig 1, annotators provided the lowest ratings for all three characteristics, while the loudness algorithm rated the environment as being louder than the two human sources. Annotators rarely rated brightness and loudness levels using the maximum level of ‘4’, resulting in narrower and lower average ratings than those of the participants (Fig 1). Similarly, the brightness algorithm never attributed the maximum score for brightness levels. Regarding attendance, participants and the computer vision algorithm reported the presence of six people on average, while annotators reported an average of five. For each characteristic, ratings from the three sources were all positively correlated (Fig 1). Yet, the level of agreement between participants and both annotators and algorithms was lower (*r* = .21 to .57; all *p* < .001) than between annotators and algorithms (*r* = .62 to .82; all *p* < .001).

**Table 1:** Average ratings of brightness, loudness and number of people per data source, and bivariate correlations with drinking behaviors and participants’ demographics.

	Brightness	Loudness	Attendance
Scale	0–4	0–4	Linear
Participants’ ratings in situ, mean (SD)	2.0 (1.2) ^b^	1.7 (1.4) ^b^	6.2 (8.9) ^b^
Annotators’ ratings from videos, mean (SD)	1.5 (0.9) ^a^	1.6 (1.1) ^a^	5.0 (5.2) ^a^
Algorithms, mean (SD)	1.9 (1.0) ^b^	2.3 (1.1) ^c^	6.1 (9.7) ^b^
Pearson’s correlations (*r*, *p*-value)			
Participants’ ratings:			
Brightness	-	-.01, p = .683	.04, p = .581
Loudness	-	-	.35, p < .001
Current alcoholic drink (yes = 1)	-.06, p = .008	.33, p < .001	.21, p < .001
Number of earlier alcoholic drinks	-.05, p = .130	.22, p = .002	.14, p = .062
Annotators’ ratings:			
Brightness	-	-.27, p < .001	-.06, p = .310
Loudness	-	-	.63, p < .001
Current alcoholic drink (yes = 1)	-.23, p < .001	.47, p < .001	.30, p < .001
Number of earlier alcoholic drinks	-.14, p = .005	.20, p = .001	.16, p = .008
Computer analysis ratings:			
Brightness	-	-.31, p < .001	.14, p = .001
Loudness	-	-	.34, p < .001
Current alcoholic drink (yes = 1)	-.27, p < .001	.44, p < .001	.16, p < .001
Number of earlier alcoholic drinks	-.12, p = .034	.19, p = .001	.05, p = .108

Note: N = 825; paired-sample *t*-tests in columns with a < b < c at p < .05 significance level.

For all sources, loudness was positively correlated with attendance (*r* = .34 to .63; all *p* < .001; Table 1). In addition, based on annotators’ and algorithms’ ratings, brightness and loudness were negatively correlated (*r* = -.27 and -.31; all *p* < .001). Concurrent alcohol use was positively correlated with loudness and attendance for all sources (*r* = .16 to .47; all *p* < .001), and negatively correlated with brightness for annotators (*r* = -.23; *p* < .001) and algorithms (*r* = -.27; *p* < .001). Overall, patterns of ratings appear very similar across all three sources, and especially between annotators and algorithms.

### Variations across location types and alcohol use

As shown in Table 2, the average levels of brightness, loudness, and attendance varied across types of locations (see superscript letters indicating mean differences in each column). For example, all three sources rated restaurants, modes of transport, and events among the brightest environments, while public parks and nightclubs were rated among the darkest. Nightclubs, events, and bars were rated among the loudest environments, and private places as the most quiet. Lastly, nightclubs, events, bars, and restaurants were rated as the most attended places, whereas private places were the least attended. Interestingly, in dark environments the algorithm detected a lower number of people than the participants, highlighting the difficulty the algorithm had in detecting shapes in dark videos.

**Table 2:** Number of situations, and levels of brightness, loudness and attendance, per data source, location type, and type of drink consumed (alcoholic vs. non-alcoholic).

		Number of observations		Brightness			
		Participants	Annotators	Participants	Annotators	Algorithm	
		N (%)	N (%)	Mean (SD)	Mean (SD)	Mean (SD)	
Bars / Pubs							
	Total	345	124	1.7 (1.1) ^a^	1.0 (0.7) ^b^	1.3 (0.8) ^b^	
	Non-alc. drinks	23 (6.7%)	10 (8.1%)	1.5 (1.3)	0.6 (0.8)	0.6 (0.8)	
	Alcoholic drinks	322 (93.3%)	114 (91.9%)	1.7 (1.0)	1.1 (0.6)	1.3 (0.8)	
	Cohen’s *d*			0.19	0.70	0.87	
	Cohen’s *d* 95%-CI			(-0.23, 0.61)	(0.05, 1.35)	(0.21, 1.52)	
Nightclubs							
	Total	86	43	1.5 (1.2) ^a^	0.6 (0.7) ^a^	0.9 (0.8) ^a^	
	Non-alc. drinks	5 (5.8%)	4 (9.3%)	2.4 (1.5)	1.0 (0.8)	1.3 (0.5)	
	Alcoholic drinks	81 (94.2%)	39 (90.7%)	1.4 (1.2)	0.6 (0.7)	0.8 (0.8)	
	Cohen’s *d*			-0.83	-0.63	-0.56	
	Cohen’s *d* 95%-CI			(-1.75, 0.08)	(-1.67, 0.41)	(-1.59, 0.48)	
Restaurants							
	Total	129	42	2.4 (1.0) ^b^	2.2 (0.7) ^d^	2.5 (0.6) ^c^	
	Non-alc. drinks	64 (49.6%)	22 (52.4%)	2.3 (1.1)	2.3 (0.8)	2.6 (0.6)	
	Alcoholic drinks	65 (50.4%)	20 (47.6%)	2.4 (1.0)	2.0 (0.6)	2.3 (0.7)	
	Cohen’s *d*			0.04	-0.44	-0.54	
	Cohen’s *d* 95%-CI			(-0.31, 0.38)	(-1.05, 0.17)	(-1.16, 0.07)	
Events / Other commercial							
	Total	74	41	2.3 (1.2) ^b^	2.1 (0.8) ^d^	2.3 (0.8) ^c^	
	Non-alc. drinks	20 (27.0%)	9 (22.0%)	2.1 (1.1)	1.9 (1.1)	2.0 (0.9)	
	Alcoholic drinks	54 (73.0%)	32 (78.0%)	2.4 (1.2)	2.1 (0.8)	2.4 (0.7)	
	Cohen’s *d*			0.32	0.28	0.58	
	Cohen’s *d* 95%-CI			(-0.20, 0.83)	(-0.47, 1.02)	(-0.17, 1.33)	
Streets / Parks							
	Total	324	127	1.6 (1.2) ^a^	0.8 (0.6) ^a^	1.1 (0.9) ^a,b^	
	Non-alc. drinks	41 (12.7%)	16 (12.6%)	1.8 (1.1)	0.9 (0.7)	1.1 (0.8)	
	Alcoholic drinks	283 (87.3%)	111 (87.4%)	1.5 (1.2)	0.7 (0.6)	1.1 (0.9)	
	Cohen’s *d*			-0.22	-0.33	0.02	
	Cohen’s *d* 95%-CI			(-0.55, 0.11)	(-0.85, 0.20)	(-0.50, 0.55)	
Travelling							
	Total	114	64	2.4 (1.3) ^b^	2.1 (0.8) ^d^	2.4 (0.8) ^c^	
	Non-alc. drinks	32 (28.1%)	19 (29.7%)	2.2 (1.0)	1.9 (0.9)	2.3 (1.0)	
	Alcoholic drinks	82 (71.9%)	45 (70.3%)	2.5 (1.4)	2.2 (0.8)	2.4 (0.8)	
	Cohen’s *d*			0.22	0.35	0.21	
	Cohen’s *d* 95%-CI			(-0.19, 0.63)	(-0.19, 0.89)	(-0.32, 0.75)	
Private places							
	Total	1286	384	2.2 (1.2) ^b^	1.8 (0.7) ^c^	2.3 (0.9) ^c^	
	Non-alc. drinks	601 (46.7%)	189 (49.2%)	2.1 (1.1)	1.9 (0.6)	2.5 (0.7)	
	Alcoholic drinks	685 (53.3%)	195 (50.8%)	2.3 (1.2)	1.7 (0.8)	2.1 (1.0)	
	Cohen’s *d*			0.09	-0.29	-0.40	
	Cohen’s *d* 95%-CI			(-0.02, 0.20)	(-0.49, -0.09)	(-0.61, -0.20)	
		Loudness			Attendance		
		Participants	Annotators	Algorithm	Participants	Annotators	Algorithm
		Mean (SD)	Mean (SD)	Mean (SD)	Mean (SD)	Mean (SD)	Mean (SD)
Bars / Pubs						
	Total	2.5 (1.2) ^c^	2.5 (0.6) ^d^	3.1 (0.5) ^c^	7.1 (8.8) ^c^	8.5 (5.0) ^c^	7.1 (8.4) ^c^
	Non-alc. drinks	2.0 (1.0)	2.4 (0.5)	3.3 (0.5)	10.9 (16.6)	9.1 (5.3)	5.9 (8.9)
	Alcoholic drinks	2.5 (1.2)	2.5 (0.6)	3.1 (0.5)	6.8 (7.9)	8.4 (5)	7.2 (8.4)
	Cohen’s *d*	0.50	0.10	-0.32	-0.46	-0.13	0.15
	Cohen’s *d* 95%-CI	(0.07, 0.92)	(-0.55, 0.74)	(-0.97, 0.32)	(-0.89, -0.04)	(-0.78, 0.51)	(-0.50, 0.80)
Nightclubs						
	Total	2.7 (1.5) ^c,d^	3.0 (0.8) ^e^	3.5 (0.6) ^d^	10.2 (9.5) ^d^	10.5 (5.9) ^d^	7.3 (8.8) ^c,d^
	Non-alc. drinks	3.4 (0.9)	3.3 (0.5)	3.5 (0.6)	14 (10.7)	12.9 (4.2)	12.5 (12)
	Alcoholic drinks	2.6 (1.5)	2.9 (0.8)	3.5 (0.6)	10 (9.4)	10.3 (6)	6.8 (8.4)
	Cohen’s *d*	-0.53	-0.37	-0.06	-0.42	-0.44	-0.65
	Cohen’s *d* 95%-CI	(-1.44, 0.37)	(-1.41, 0.66)	(-1.09, 0.97)	(-1.33, 0.49)	(-1.47, 0.60)	(-1.69, 0.38)
Restaurants						
	Total	1.9 (1.2) ^b^	1.9 (0.7) ^c^	2.7 (0.5) ^b^	5.3 (7.1) ^a,b^	8.1 (4.4) ^c^	10.4 (8.1) ^d^
	Non-alc. drinks	1.7 (1.2)	1.8 (0.8)	2.7 (0.5)	5.9 (8)	8 (4.7)	9.6 (8)
	Alcoholic drinks	2.1 (1.2)	2.0 (0.5)	2.7 (0.5)	4.7 (6)	8.1 (4.1)	11.3 (8.4)
	Cohen’s *d*	0.32	0.28	-0.07	-0.18	0.03	0.20
	Cohen’s *d* 95%-CI	(-0.03, 0.66)	(-0.33, 0.89)	(-0.67, 0.54)	(-0.53, 0.17)	(-0.58, 0.63)	(-0.40, 0.81)
Events / Other commercial							
	Total	2.9 (1.0) ^d^	2.6 (0.8) ^d^	3.1 (0.5) ^c^	14.2 (12.2) ^e^	12.3 (3.9) ^d^	24.7 (24.5) ^e^
	Non-alc. drinks	2.8 (1.1)	2.1 (0.8)	2.9 (0.6)	9.6 (9.1)	9.3 (4.7)	11.4 (8.8)
	Alcoholic drinks	2.9 (0.9)	2.8 (0.7)	3.1 (0.4)	15.9 (12.8)	13.1 (3.2)	28.4 (26.2)
	Cohen’s *d*	0.20	0.87	0.51	0.53	1.07	0.71
	Cohen’s *d* 95%-CI	(-0.31, 0.72)	(0.11, 1.64)	(-0.24, 1.26)	(0.01, 1.05)	(0.29, 1.84)	(-0.04, 1.47)
Streets / Parks						
	Total	1.8 (1.4) ^b^	1.7 (0.8) ^c^	2.6 (0.6) ^b^	5.9 (6.7) ^b,c^	4.5 (4.1) ^b^	5.0 (8.0) ^b^
	Non-alc. drinks	1.7 (1.3)	1.4 (1.0)	2.4 (0.5)	5.1 (7.1)	4.5 (4.4)	6.4 (12.6)
	Alcoholic drinks	1.8 (1.4)	1.8 (0.7)	2.6 (0.6)	6 (6.6)	4.5 (4.1)	4.8 (7.1)
	Cohen’s *d*	0.07	0.45	0.21	0.12	0.02	-0.21
	Cohen’s *d* 95%-CI	(-0.26, 0.40)	(-0.07, 0.98)	(-0.32, 0.73)	(-0.20, 0.45)	(-0.51, 0.54)	(-0.74, 0.31)
Travelling						
	Total	1.7 (1.3) ^b^	1.4 (0.8) ^b^	2.6 (0.7) ^b^	3.1 (4.6) ^a^	5.6 (4.7) ^b^	8.5 (10.0) ^c,d^
	Non-alc. drinks	1.8 (1.2)	1.2 (0.9)	2.5 (0.7)	2 (3.5)	5.9 (5.9)	9.7 (14.2)
	Alcoholic drinks	1.6 (1.3)	1.5 (0.8)	2.6 (0.7)	3.5 (5)	5.5 (4.2)	8 (7.8)
	Cohen’s *d*	-0.2	0.41	0.07	0.33	-0.09	-0.17
	Cohen’s *d* 95%-CI	(-0.61, 0.21)	(-0.13, 0.95)	(-0.46, 0.61)	(-0.08, 0.74)	(-0.63, 0.44)	(-0.71, 0.37)
Private places						
	Total	1.3 (1.3) ^a^	1.0 (1.0) ^a^	1.7 (1.1) ^a^	5.9 (8.8) ^b^	2.3 (3.4) ^a^	3.2 (4.3) ^a^
	Non-alc. drinks	0.8 (1.1)	0.5 (0.8)	1.1 (1.0)	2.9 (5.5)	0.9 (2.4)	1.7 (2.9)
	Alcoholic drinks	1.7 (1.2)	1.6 (1.0)	2.2 (1.0)	8.5 (10.3)	3.6 (3.7)	4.6 (4.9)
	Cohen’s *d*	0.80	1.21	1.04	0.67	0.87	0.72
	Cohen’s *d* 95%-CI	(0.69, 0.91)	(0.99, 1.43)	(0.83, 1.26)	(0.56, 0.78)	(0.66, 1.08)	(0.51, 0.92)

Note: a-e) *t*-tests in columns with *a* < *b* < *c* < *d* < *e* at p < .05 significance level.

Table 2 also shows the average levels of brightness, loudness, and attendance per type of location depending on whether participants documented an alcoholic or a non-alcoholic drink. Nearly all drinks reported in nightclubs (94.2%), pubs (93.3%), and public streets and parks (86.9%) contained alcohol. Inversely, only half of the drinks reported in private places (52.9%) and restaurants (50%) contained alcohol. Overall, similar variations in contextual characteristic levels between situations with and without alcohol use could be observed for all sources (e.g., all sources rated nightclubs as being darker, louder, and less attended when alcohol was consumed) but most effects were either of small (absolute value of Cohen’s *d* under 0.2) to medium magnitude (under 0.5) or were not significant (Cohen’s *d* 95%-confidence interval contains ‘0’). Nevertheless, a couple of noteworthy effects can be observed. First, all sources rated private places as much louder (+1 on the 5-point scale; *d* = 0.80 to 1.21) and much more crowded (about 3 times more people; *d* = 0.67 to 0.87) when alcohol was consumed. Additionally, private places were rated as darker by annotators (*d* = -0.29) and the algorithm (*d* = -.0.40) when alcohol was consumed. Regarding bars/pubs, annotators (*d* = 0.70) and the algorithm (*d* = 0.87) rated the location as being brighter, and participants as being louder (*d* = 0.50) when alcohol was consumed. Lastly, annotators rated events as being louder (*d* = 0.87) and more attended (*d* = 1.07) when alcohol was consumed. Unsurprisingly, the high attendance at festivals, concerts, or sporting events resulted in a particularly high number of people identified by the algorithm.

### Mutlivariate associations of the context with alcohol use

Table 3 presents the extent to which variations in brightness, loudness, and attendance, perceived by either participants, annotators, or computer algorithms are associated with the consumption of an alcoholic drink (versus a non-alcoholic drink, as the reference category) in the three major types of nightlife settings. In commercial venues, results show that participants were more likely to drink alcohol when the context was reported as being less bright by all three sources (OR_participants_ = 0.67 [CI: 0.47–0.94]; OR_annotators_ = 0.53 [CI: 0.33–0.85]; OR_algorithm_ = 0.58 [CI: 0.37–0.88]) and louder by participants (OR = 1.40 [CI: 1.02–1.92]) and annotators (OR = 2.45 [CI: 1.25–4.80]). In public spaces, no clear association of alcohol use with brightness, loudness and attendance levels was found for all three sources. Finally, the likelihood of drinking alcohol in private places was associated with all three investigated contextual characteristics. In private, alcohol use was more likely with increased loudness according to all three sources (OR_participants_ = 1.74 [CI: 1.31–2.32]; OR_annotators_ = 3.22 [CI: 2.06–5.03]; OR_algorithm_ = 2.62 [CI: 1.83–3.76]), and with higher attendance according to the participants (OR = 1.10 [CI: 1.03–1.18]) and the algorithm (OR = 1.19 [CI: 1.07–1.32]), and with reduced brightness according to the algorithm (OR = 0.64 [CI: 0.44–0.94]). No effect of the number of prior drinks consumed was found.

**Table 3:** Multivariate associations of contextual characteristics with the likelihood of consuming alcoholic drinks in different settings, separately per data source, and models performance indices.

	Commercial venues (e.g., pubs, nightclubs)			Public spaces (e.g., streets, parks, travelling)			Private places (e.g., homes)		
	Participants	Annotators	Algorithms	Participants	Annotators	Algorithms	Participants	Annotators	Algorithms
	OR (95%-CI)	OR (95%-CI)	OR (95%-CI)	OR (95%-CI)	OR (95%-CI)	OR (95%-CI)	OR (95%-CI)	OR (95%-CI)	OR (95%-CI)
Situation level									
Brightness	0.67 (0.47–0.94)	0.53 (0.33–0.85)	0.58 (0.37–0.88)	0.66 (0.39–1.13)	0.60 (0.30–1.19)	0.69 (0.38–1.25)	0.96 (0.73–1.26)	0.83 (0.55–1.27)	0.64 (0.44–0.94)
Loudness	1.40 (1.02–1.92)	2.45 (1.25–4.80)	1.39 (0.69–2.80)	0.61 (0.34–1.11)	2.06 (0.97–4.35)	1.16 (0.46–2.87)	1.74 (1.31–2.32)	3.22 (2.06–5.03)	2.62 (1.83–3.76)
Attendance	1.00 (0.96–1.03)	0.97 (0.89–1.06)	1.03 (0.99–1.06)	1.18 (1.00–1.39)	0.91 (0.79–1.05)	0.96 (0.90–1.02)	1.10 (1.03–1.18)	1.14 (0.99–1.33)	1.19 (1.07–1.32)
Prior drinks	1.14 (0.97–1.34)	1.10 (0.93–1.30)	1.15 (0.98–1.35)	1.11 (0.88–1.40)	1.13 (0.90–1.40)	1.12 (0.91–1.39)	1.02 (0.91–1.13)	1.02 (0.91–1.14)	1.03 (0.91–1.17)
Variance explained									
McKelvey & Zavoina’s R^2^	0.17	0.25	0.17	0.34	0.23	0.15	0.34	0.42	0.46
Goodness of fit									
Hosmer-Lemeshow χ^2^	1.89, p = .984	2.63, p = .955	2.19, p = .975	3.96, p = .861	2.51, p = .961	3.84, p = .871	6.73, p = .565	4.29, p = .830	13.49, p = .096
Cross-validation task									
Inference accuracy	0.69	0.84	0.79	0.71	0.82	0.86	0.69	0.80	0.76

Note: N = 825; CI = confidence interval.

### Models fit and inference accuracy

Table 3 also presents the percentage of variance explained, the goodness of fit, and the inference accuracy for each of the nine logistic regression models. For all models, the *p*-value of the Hosmer-Lemeshow test was non-significant (*p* >.05) showing no evidence of poor fit. Regarding the percentage of variance explained, contextual variables explained more variance in private places (R-squared between 0.34 and 0.46) than in the two other location types regardless of the data source type. Yet, no data source seemed to outperform the others overall since annotators’ reports explained slightly more variance in commercial venues than the two other sources, while it was the case for participants’ ratings in public spaces and for algorithms’ ratings in private places. Lastly, results of the cross-validation task showed that the inference accuracies of the models based on the annotators’ ratings (80% to 84%) and the algorithms’ ratings (76% to 86%) were higher than those of the models based on the participants’ ratings (69% to 71%).

## Discussion

The overall purpose of this paper was to investigate how a select set of contextual characteristics assessed by the in-situ actors, external human observers (annotators), and computer algorithms are associated with the consumption of alcohol in different nightlife settings. Data were collected by means of a custom-built smartphone application recording event-contingent reports of alcohol use, location attended, brightness, loudness and attendance levels from study participants, as well as 10-second panoramic video clips of the drinking environment. Because videos were recorded at the same time as drinks were consumed, contextual characteristics could then be assessed by external annotators and computer algorithms with the same level of ecological validity.

### Which data source to favor

Given the unique opportunity to compare three measures of the same contextual characteristics from different data sources, one study aim was to explore the specificities of the data collected from each source and their ability to predict participants’ choice to drink alcohol or not, in general and in different types of locations. Alongside the development of smartphones and sensors to unobtrusively collect data on people’s real-life behaviors [4, 5], this study explored whether the participants’ reports of the context can be replaced by similar measures from other sources.

Differences in raw ratings (e.g., the algorithm attributed higher loudness levels than the other sources) show that neither algorithms (despite calibrating brightness and loudness levels to match human perception abilities), nor external annotators can effectively substitute for the actors’ in-situ experience in absolute terms. For instance, unlike participants, annotators and the algorithm almost never selected the highest score for brightness. Additionally, the algorithm almost exclusively rated nightclubs, streets, and parks as dark or very dark, while several participants reported them as being relatively bright. This discrepancy can partly be explained by the different ways smartphone cameras and human eyes function: camera sensors adapt by increasing contrast in very bright environments (so that videos appear moderately bright) [59] and the human eye adapts to dark conditions [60]. Similarly, the finding that the algorithm tended to rate the context as louder than humans (participants and annotators) might be explained by the fact that the audio sensors capture and account for very high energy sounds that cannot be perceived by humans. Differences between participants’ and observers’ raw ratings may also result from the conditions in which participants recorded the videos. Participants may have provided biased representations of the situation by, for example, standing close to loudspeakers, talking while recording in a silent place, or recording videos in another place than the one described in the in-situ questionnaire (e.g., chill-out room of a nightclub). They might also have not complied with the instructions on how to record the video, e.g., by failing to record a 360-degree panorama or focusing more on bright or dark zones. Occasional lack of compliance to instructions appears likely, given the high consistency between annotators’ and algorithms’ ratings.

However, above and beyond these differences in raw ratings, the patterns of bivariate associations across contextual characteristics and the ratings order per location type (e.g., loudness: nightclubs > pubs > travelling > private places) were similar for all three sources. These results suggest that, in relative terms, videos captured relevant variations of contextual characteristics in a similar way as was experienced by the participants. The three data sources can thus be considered as being partly interchangeable for correlational and regression analyses.

Regarding external validity, results of the inference task, which estimated the ability of any random subset of observations to replicate the findings of the rest of the sample, revealed higher inference accuracies for the models based on the annotators’ and on the algorithms’ ratings than those based on the participants’ ratings. This result highlights the utility of annotations and of algorithmic analyses to obtain measures of the context that are as standardized as possible, notably by having multiple rounds of annotations with each annotator working on the entire set of videos. Their ratings are therefore less fluctuant, or more predictable, than those of participants. Yet, this does not mean that annotators’ and the algorithms’ ratings would necessarily perform better at predicting alcohol use. In fact, results showed that annotators’ and the algorithms’ ratings explained more variance in private places and in commercial venues, but that participants’ ratings explained more variance in outdoor locations. In the present case, in line with the finding that contextual characteristics were weakly associated with alcohol use in public spaces, these results suggest that the choice of drinking alcohol in such locations might rather relate to other factors (e.g., night-level drinking intentions [61], off-licensed alcohol prices [62]) that could be better reported subjectively by the participants than sensed by observers.

These results have important implications for future research on context-dependent behaviors. Unless researchers are primarily interested in raw ratings and despite the fact that annotators or algorithms perform slightly worse in some locations, future studies are recommended to collect contextual data via sensors or media files whenever possible. This would reduce participant burden [63], and save participants’ time for reporting impressions, cognitions or other momentary circumstances that cannot be documented in another way. Additionally, the high correlations between annotators’ and algorithms’ ratings, as well as the high inference accuracy scores obtained for algorithms’ ratings, suggest that part of the research cost, burden, and privacy issues may be alleviated by using algorithmic analyses for extracting basic information from videos rather than annotators.

### Contextual correlates of alcohol use

A second aim of the study was to investigate how variations in brightness, loudness, and attendance identified by the participants, annotators, and algorithms relate to whether alcohol is consumed. The results notably corroborate previous evidence showing that increased odds of drinking alcohol are associated with larger numbers of people present based on participants’ reports [12, 61, 64], as well as with higher levels of loudness in pubs and nightclubs from an external observer’s perspective [21, 24]. Although the study design does not allow us to determine whether participants choose to attend darker venues for drinking or whether changes in the venues context influenced the choice to order an alcoholic drink, the consistent associations between characteristics of the physical context and alcohol use might have implications for public health. In the same way that alcohol use was experimentally proven to increase with music loudness levels [23, 24], this study suggests that manipulating the brightness level might influence alcohol use. Therefore, similarly to policies regulating maximum loudness levels in nightclubs and events to prevent hearing loss [65], minimum brightness levels might be implemented and evaluated to determine whether those constitute an effective structural prevention measure to reduce alcohol intoxication and related harms.

The present study also extends the existing literature by providing detailed and systematic results on contextual characteristics outside of commercial venues. Among all location types investigated, the interplay between contextual characteristics and drinking was particularly evident in private places. Qualitative research provided convergent indications that young people can influence their experience of drunkenness by staging the atmosphere (e.g., music types and volume, lighting) in private places [16, 17, 66]. Yet, such evidence had not been replicated in quantitative research because private places only recently became accessible for large-scale in-situ observations and measurements thanks to the growth of smartphone ownership. The findings of this study that private places were louder (and darker according to algorithms) when alcohol was consumed, confirms previous evidence that people prepare their homes when drinking on a weekend night, e.g., by moving furniture, manipulating lighting, and changing the music type and volume. In fact, unlike commercial venues, private settings can be configured by their users by manipulating the attributes of the place depending on the number of attendees and the planned activities (music selection, leaving enough space for socializing, etc.) [67, 68].

### Limitations and strengths

Several limitations of the current analyses should be acknowledged. Firstly, results do not provide evidence on whether changes in the investigated contextual characteristics directly increase the likelihood of drinking, or are the consequence of it, and should not be interpreted as causal relationships. The present results need replication in controlled conditions to better understand, at the individual level, if people choose to enter venues with specific contextual characteristics with the intention to drink alcohol, and, at the venue level, which contextual characteristics can promote or prevent alcohol use [24]. Secondly, a large number of analyzes were carried out in order to compare the levels of the three contextual characteristics across three sources, in multiple location types, and in two types of situations (with and without alcohol). The purpose of presenting all these results was to provide a complete overview of the differences across sources for future studies. This, however, bears the risk of an increased type 1 error. The present results should thus be considered as mainly exploratory and require replication. Thirdly, annotators worked in uncontrolled conditions, probably with self-set screen brightness settings and audio rendering devices. This may have caused some variations in conditions between annotation sessions within and between annotators. Fourthly, the study focused on contextual characteristics that could reliably be annotated by external observers and identified by computer algorithms from a short video clip. This approach de facto excluded many aspects that have also been shown to influence drinking behaviors at the event level [3, 15], such as motives and expectancies [29, 30], drinking of peers [69], activities done while drinking (e.g. chatting, watching TV, playing games) [70], or the gender composition of the drinking group [71], but that could not be identified by computer algorithms.

While questionnaire-based ecological momentary assessment studies generally request participants document their behavior within several minutes or hours [15, 72, 73], a major asset of recording contexts in videos is to force participants to provide an instant snapshot of the momentary circumstances. Thus, this study has the advantage of collecting behavioral and contextual data at the very *event* level, namely, the exact same time and space as the event of interest, enhancing, therefore, the ecological and internal validity of the findings. Yet, qualitative feedback after the seventh week of the app-fieldwork revealed that recording panoramic videos clips was not an ordinary action for young adults on weekend nights [74]. While recording selfies might be common on nights out, some participants may find intentionally filming the location and the people present intrusive and burdensome [28]. This might explain why only 210 out of the 241 study participants documented their drinking events with pictures and videos. To keep response burden as low as possible, we requested participants, for example, take videos only when they changed location rather than for every drink consumed. Future research should also consider the balance between data quantity and participant burden [28].

## Conclusions

This study explored the feasibility of collecting diverse data on the physical and social characteristics of drinking occasions at the event level, and examined how contextual characteristics, assessed by either participants, annotators or computer algorithms, relate to alcohol use. The results showed that this could reliably be achieved by requesting participants record a 10-second video clip of their context whenever they had a drink, and annotate those using either human annotators or algorithms. In terms of methods, this study showed that, despite differences in raw ratings, annotators’ or algorithms’ ratings might serve as substitute to participants’ in-situ impressions for correlational and regression analyses, and offer a higher degree of replicability of the findings. In terms of public health, findings that the consumption of alcohol in private places and in commercial venues is associated with reduced brightness and increased loudness might serve as a foundation for the development and evaluation of structural prevention measures to reduce alcohol intoxication and related harms.

## References

[1] Burke NJ, Joseph G, Pasick RJ, Barker JC. Theorizing Social Context: Rethinking Behavioral Theory. Health Educ Behav. 2009;36: 55S–70S. 10.1177/1090198109335338 19805791 PMC3548316

[2] McCarty D. Environmental factors in substance abuse. Determinants of substance abuse. Springer; 1985. pp. 247–281.

[3] Stanesby O, Labhart F, Dietze P, Wright CJC, Kuntsche E. The contexts of heavy drinking: A systematic review of the combinations of context-related factors associated with heavy drinking occasions. Francis JM, editor. PLOS ONE. 2019;14: e0218465. 10.1371/journal.pone.0218465 31291261 PMC6619678

[4] Santani D, Do TMT, Labhart F, Landolt S, Kuntsche E, Gatica-Perez D. DrinkSense: Characterizing Youth Drinking Behavior using Smartphones. IEEE Trans Mob Comput. 2018;17: 1536–1233. 10.1109/TMC.2018.2797901

[5] Santani D, Biel J-I, Labhart F, Truong J, Landolt S, Kuntsche E, et al. The night is young: urban crowdsourcing of nightlife patterns. Proceedings of the 2016 ACM International Joint Conference on Pervasive and Ubiquitous Computing. ACM Press; 2016. pp. 427–438. 10.1145/2971648.2971713

[6] Letherby G, Scott J, Williams M. Objectivity and subjectivity in social research. Sage; 2012.

[7] Merton RK. Insiders and Outsiders: A Chapter in the Sociology of Knowledge. Am J Sociol. 1972;78: 9–47. 10.1086/225294

[8] Olson DH. Insiders’ and outsiders’ views of relationships: Research studies. Close Relatsh Perspect Mean Intim. 1977; 115–135.

[9] Bertholet N, Faouzi M, Studer J, Daeppen J-B, Gmel G. Perception of tobacco, cannabis, and alcohol use of others is associated with one’s own use. Addict Sci Clin Pract. 2013;8. 10.1186/1940-0640-8-8 24499600 PMC3853223

[10] Demers A, Kairouz S, Adlaf E, Gliksman L, Newton-Taylor B, Marchand A. Multilevel analysis of situational drinking among Canadian undergraduates. Soc Sci Med. 2002;55: 415–424. 10.1016/s0277-9536(01)00258-1 12144149

[11] Thrul J, Lipperman-Kreda S, Grube JW. Do Associations Between Drinking Event Characteristics and Underage Drinking Differ by Drinking Location? J Stud Alcohol Drugs. 2018;79: 417–422. 10.15288/jsad.2018.79.417 29885149 PMC6005261

[12] Thrul J, Kuntsche E. The impact of friends on young adults’ drinking over the course of the evening-an event-level analysis: Impact of friends on young adults’ drinking. Addiction. 2015;110: 619–626. 10.1111/add.12862 25732756

[13] Smit K, Groefsema M, Luijten M, Engels R, Kuntsche E. Drinking Motives Moderate the Effect of the Social Environment on Alcohol Use: An Event-Level Study Among Young Adults. J Stud Alcohol Drugs. 2015;76: 971–980. 10.15288/jsad.2015.76.971 26562607

[14] Labhart F, Graham K, Wells S, Kuntsche E. Drinking Before Going to Licensed Premises: An Event-Level Analysis of Predrinking, Alcohol Consumption, and Adverse Outcomes. Alcohol Clin Exp Res. 2013;37: 284–291. 10.1111/j.1530-0277.2012.01872.x 23136847

[15] Stevely AK, Holmes J, Meier PS. Contextual characteristics of adults’ drinking occasions and their association with levels of alcohol consumption and acute alcohol‐related harm: a mapping review. Addiction. 2019;115: 218–229. 10.1111/add.14839 31655026

[16] Wilkinson S. Drinking in the dark: shedding light on young people’s alcohol consumption experiences. Soc Cult Geogr. 2017;18: 739–757. 10.1080/14649365.2016.1227872

[17] Bille M. Lighting up cosy atmospheres in Denmark. Emot Space Soc. 2015;15: 56–63. 10.1016/j.emospa.2013.12.008

[18] Coughlin SS. Recall bias in epidemiologic studies. J Clin Epidemiol. 1990;43: 87–91. 10.1016/0895-4356(90)90060-3 2319285

[19] Ekholm O. Influence of the recall period on self-reported alcohol intake. Eur J Clin Nutr. 2004;58: 60–63. 10.1038/sj.ejcn.1601746 14679368

[20] Graham K, Bernards S, Osgood DW, Wells S. Bad nights or bad bars? Multi-level analysis of environmental predictors of aggression in late-night large-capacity bars and clubs. Addiction. 2006;101: 1569–1580. 10.1111/j.1360-0443.2006.01608.x 17034436

[21] Hughes K, Quigg Z, Eckley L, Bellis M, Jones L, Calafat A, et al. Environmental factors in drinking venues and alcohol-related harm: the evidence base for European intervention: Drinking venues and alcohol-related harm. Addiction. 2011;106: 37–46. 10.1111/j.1360-0443.2010.03316.x 21324020

[22] Miller P, Pennay A, Jenkinson R, Droste N, Chikritzhs T, Tomsen S, et al. Patron offending and intoxication in night-time entertainment districts (POINTED): A study protocol. Int J Alcohol Drug Res. 2013;2: 69–76. 10.7895/ijadr.v2i1.74

[23] Guéguen N, Hélène LG, Jacob C. Sound Level of Background Music and Alcohol Consumption: An Empirical Evaluation. Percept Mot Skills. 2004;99: 34–38. 10.2466/pms.99.1.34-38 15446627

[24] Guéguen N, Jacob C, Le Guellec H, Morineau T, Lourel M. Sound Level of Environmental Music and Drinking Behavior: A Field Experiment With Beer Drinkers. Alcohol Clin Exp Res. 2008;32: 1795–1798. 10.1111/j.1530-0277.2008.00764.x 18647281

[25] Welch D, Fremaux G. Why Do People Like Loud Sound? A Qualitative Study. Int J Environ Res Public Health. 2017;14: 908. 10.3390/ijerph14080908 28800097 PMC5580611

[26] Forsyth A, Cloonan M. Alco‐pop? The Use of Popular Music in Glasgow Pubs. Pop Music Soc. 2008;31: 57–78. 10.1080/03007760601061902

[27] Hughes K, Quigg Z, Bellis MA, Calafat A, Hasselt N van, Kosir M, et al. Drunk and Disorganised: Relationships between Bar Characteristics and Customer Intoxication in European Drinking Environments. Int J Environ Res Public Health. 2012;9: 4068–4082. 10.3390/ijerph9114068 23202832 PMC3524613

[28] Labhart F, Tarsetti F, Bornet O, Santani D, Truong J, Landolt S, et al. Capturing drinking and nightlife behaviours and their social and physical context with a smartphone application–investigation of users’ experience and reactivity. Addict Res Theory. 2020;28: 62–75. 10.1080/16066359.2019.1584292

[29] Stevens AK, Haikalis M, Merrill JE. Unplanned vs. planned drinking: Event-level influences of drinking motives and affect. Addict Behav. 2021;112: 106592. 10.1016/j.addbeh.2020.106592 32768795 PMC7572627

[30] Lau-Barraco C, Braitman AL, Linden-Carmichael AN, Stamates AL. Differences in weekday versus weekend drinking among nonstudent emerging adults. Exp Clin Psychopharmacol. 2016;24: 100–109. 10.1037/pha0000068 26901592 PMC4828908

[31] Jackson KM, Merrill JE, Barnett NP, Colby SM, Abar CC, Rogers ML, et al. Contextual influences on early drinking: Characteristics of drinking and nondrinking days. Psychol Addict Behav. 2016;30: 566–577. 10.1037/adb0000184 27269292 PMC5102807

[32] Lipperman-Kreda S, Finan LJ, Grube JW. Social and situational characteristics associated with adolescents’ drinking at party and non-party events. Addict Behav. 2018;83: 148–153. 10.1016/j.addbeh.2017.12.001 29249280 PMC5963966

[33] Hughes K, Anderson Z, Morleo M, Bellis MA. Alcohol, nightlife and violence: the relative contributions of drinking before and during nights out to negative health and criminal justice outcomes. Addiction. 2008;103: 60–65. 10.1111/j.1360-0443.2007.02030.x 17996008

[34] Kuntsche E, Kuntsche S, Thrul J, Gmel G. Binge drinking: Health impact, prevalence, correlates and interventions. Psychol Health. 2017;32: 976–1017. 10.1080/08870446.2017.1325889 28513195

[35] Labhart F, Santani D, Truong J, Tarsetti F, Bornet O, Landolt S, et al. Development of the Geographical Proportional-to-size Street-Intercept Sampling (GPSIS) method for recruiting urban nightlife-goers in an entire city. Int J Soc Res Methodol. 2017;20: 721–736. 10.1080/13645579.2017.1293928

[36] Phan T-T, Labhart F, Gatica-Perez D. My Own Private Nightlife: Understanding Youth PersonalSpaces from Crowdsourced Video. Proceedings of the ACM on Human-Computer Interaction. 2019. p. 189:1–34. 10.1145/3359291.

[37] World Health Organization. International guide for monitoring alcohol consumption and related harm. 2000 [cited 20 Mar 2018]. Available: http://whqlibdoc.who.int/hq/2000/WHO_MSD_MSB_00.4.pdf.

[38] Shrout PE, Fleiss JL. Intraclass correlations: Uses in assessing rater reliability. Psychol Bull. 1979;86: 420–428. 10.1037//0033-2909.86.2.420 18839484

[39] Cicchetti DV. Guidelines, criteria, and rules of thumb for evaluating normed and standardized assessment instruments in psychology. Psychol Assess. 1994;6: 284.

[40] Bezryadin S, Bourov P, Ilinih D. Brightness Calculation in Digital Image Processing. Int Symp Technol Digit Photo Fulfillment. 2007;2007: 10–15. 10.2352/ISSN.2169-4672.2007.1.0.10

[41] Kingdom FAA. Lightness, brightness and transparency: A quarter century of new ideas, captivating demonstrations and unrelenting controversy. Vision Res. 2011;51: 652–673. 10.1016/j.visres.2010.09.012 20858514

[42] Glasser LG, McKinney AH, Reilly CD, Schnelle PD. Cube-Root Color Coordinate System. J Opt Soc Am. 1958;48: 736. 10.1364/JOSA.48.000736

[43] Gonzalez RC, Woods RE. Digital image processing. Prentice hall Upper Saddle River, NJ; 2002.

[44] Kim H-G, Moreau N, Sikora T. MPEG-7 audio and beyond: Audio content indexing and retrieval. John Wiley & Sons; 2006.

[45] Krug E, Cieza MA, Chadha S, Sminkey L, Morata T, Swanepoel D, et al. Make listening safe. Geneva World Health Organ. 2015.

[46] Berger EH, Neitzel R, Kladden CA. Noise navigatorTM sound level database with over 1700 measurement values. In: Noise navigatorTM sound level database. Version 1.8 [Internet]. 22 Aug 2016 [cited 6 Nov 2019]. Available: http://multimedia.3m.com/mws/media/1262312O/3m-noise-navigator.xlsx.

[47] Redmon J, Farhadi A. YOLOv3: An Incremental Improvement. ArXiv180402767 Cs. 2018 [cited 6 Nov 2019]. Available: http://arxiv.org/abs/1804.02767.

[48] Lin T-Y, Maire M, Belongie S, Hays J, Perona P, Ramanan D, et al. Microsoft COCO: Common Objects in Context. In: Fleet D, Pajdla T, Schiele B, Tuytelaars T, editors. Computer Vision–ECCV 2014. Cham: Springer International Publishing; 2014. pp. 740–755. 10.1007/978-3-319-10602-1_48.

[49] Wojke N, Bewley A, Paulus D. Simple online and realtime tracking with a deep association metric. 2017 IEEE International Conference on Image Processing (ICIP). Beijing: IEEE; 2017. pp. 3645–3649. 10.1109/ICIP.2017.8296962

[50] Bewley A, Ge Z, Ott L, Ramos F, Upcroft B. Simple online and realtime tracking. 2016 IEEE International Conference on Image Processing (ICIP). Phoenix, AZ, USA: IEEE; 2016. pp. 3464–3468. 10.1109/ICIP.2016.7533003

[51] Wojke N, Bewley A. Deep Cosine Metric Learning for Person Re-identification. 2018 IEEE Winter Conference on Applications of Computer Vision (WACV). Lake Tahoe, NV: IEEE; 2018. pp. 748–756. 10.1109/WACV.2018.00087

[52] Cohen J. Statistical power analysis for the behavioral sciences. Routledge; 1988.

[53] Lee DK. Alternatives to P value: confidence interval and effect size. Korean J Anesthesiol. 2016;69: 555. 10.4097/kjae.2016.69.6.555 27924194 PMC5133225

[54] Langer W. How to assess the fit of multilevel logit models with Stata? German Stata Users’ Group Meetings 2017. Stata Users Group; 2017.

[55] Schechter C. Hosmer-Lemeshow test after melogit. In: Statlist—The Stata Forum [Internet]. 2017 [cited 11 Dec 2020]. Available: https://www.statalist.org/forums/forum/general-stata-discussion/general/1392363.

[56] Chollet F. Keras. 2015. Available: https://keras.io.

[57] Chawla NV, Bowyer KW, Hall LO, Kegelmeyer WP. SMOTE: Synthetic Minority Over-sampling Technique. J Artif Intell Res. 2002;16: 321–357. 10.1613/jair.953

[58] Bishop CM. Pattern recognition and machine learning. springer; 2006.

[59] Wanat R, Mantiuk RK. Simulating and compensating changes in appearance between day and night vision. ACM Trans Graph. 2014;33: 1–12. 10.1145/2601097.2601150

[60] Winn B, Whitaker D, Elliott DB, Phillips NJ. Factors affecting light-adapted pupil size in normal human subjects. Invest Ophthalmol Vis Sci. 1994;35: 1132–1137. 8125724

[61] Labhart F, Anderson KG, Kuntsche E. The Spirit Is Willing, But the Flesh is Weak: Why Young People Drink More Than Intended on Weekend Nights-An Event-Level Study. Alcohol Clin Exp Res. 2017;41: 1961–1969. 10.1111/acer.13490 28968920

[62] Labhart F, Ferris J, Winstock A, Kuntsche E. The country-level effects of drinking, heavy drinking and drink prices on pre-drinking: An international comparison of 25 countries: Country-level effects on pre-drinking. Drug Alcohol Rev. 2017;36: 742–750. 10.1111/dar.12525 28295899

[63] Carpenter RW, Wycoff AM, Trull TJ. Ambulatory Assessment: New Adventures in Characterizing Dynamic Processes. Assessment. 2016;23: 414–424. 10.1177/1073191116632341 26887808 PMC6410721

[64] Labhart F, Wells S, Graham K, Kuntsche E. Do Individual and Situational Factors Explain the Link Between Predrinking and Heavier Alcohol Consumption? An Event-Level Study of Types of Beverage Consumed and Social Context. Alcohol Alcohol. 2014;49: 327–335. 10.1093/alcalc/agu001 24481651

[65] World Health Organization. Hearing loss due to recreational exposure to loud sounds: a review. World Health Organization; 2015. Available: https://apps.who.int/iris/bitstream/handle/10665/154589/9789241508513_eng.pdf.

[66] Pelzelmayer K, Landolt S, Truong J, Labhart F, Santani D, Kuntsche E, et al. Youth nightlife at home: Characterisations and conceptualisations of home. Child Geogr. 2021;2021: 1–12. 10.1080/14733285.2020.1718607

[67] Lincoln S. Youth culture and private space. Houndmills, Basingstoke, Hampshire; New York: Palgrave Macmillan; 2012.

[68] Labhart F, Kuntsche E. Development and validation of the predrinking motives questionnaire: Predrinking motives questionnaire. J Appl Soc Psychol. 2017;47: 136–147. 10.1111/jasp.12419

[69] Gallupe O, Bouchard M. Adolescent parties and substance use: A situational approach to peer influence. J Crim Justice. 2013;41: 162–171. 10.1016/j.jcrimjus.2013.01.002

[70] van de Goor LA, Knibbe RA, Drop MJ. Adolescent drinking behavior: an observational study of the influence of situational factors on adolescent drinking rates. J Stud Alcohol. 1990;51: 548–555. 10.15288/jsa.1990.51.548 2270064

[71] Thrul J, Labhart F, Kuntsche E. Drinking with mixed-gender groups is associated with heavy weekend drinking among young adults: Drinking groups and weekend drinking. Addiction. 2017;112: 432–439. 10.1111/add.13633 27743495 PMC5296361

[72] Kuntsche E, Labhart F. ICAT: Development of an Internet-Based Data Collection Method for Ecological Momentary Assessment Using Personal Cell Phones. Eur J Psychol Assess. 2013;29: 140–148. 10.1027/1015-5759/a000137 24285917 PMC3839619

[73] Witkiewitz K, Desai SA, Steckler G, Jackson KM, Bowen S, Leigh BC, et al. Concurrent drinking and smoking among college students: An event-level analysis. Psychol Addict Behav. 2012;26: 649–654. 10.1037/a0025363 21895348 PMC3894655

[74] Truong J, Labhart F, Santani D, Gatica-Perez D, Kuntsche E, Landolt S. The emotional entanglements of smartphones “in the field”: on social positioning, power relations, and research ethics. Area. 2019;52: 81–88. 10.1111/area.12548.

